# N-Alpha-Acetylation of α-Synuclein Increases Its Helical Folding Propensity, GM1 Binding Specificity and Resistance to Aggregation

**DOI:** 10.1371/journal.pone.0103727

**Published:** 2014-07-30

**Authors:** Tim Bartels, Nora C. Kim, Eric S. Luth, Dennis J. Selkoe

**Affiliations:** Center for Neurologic Diseases, Brigham and Women’s Hospital and Harvard Medical School, Boston, Massachusetts, United States of America; National Institute for Medical Research, Medical Research Council, London, United Kingdom

## Abstract

A switch in the conformational properties of α-synuclein (αS) is hypothesized to be a key step in the pathogenic mechanism of Parkinson’s disease (PD). Whereas the beta-sheet-rich state of αS has long been associated with its pathological aggregation in PD, a partially alpha-helical state was found to be related to physiological lipid binding; this suggests a potential role of the alpha-helical state in controlling synaptic vesicle cycling and resistance to β-sheet rich aggregation. N-terminal acetylation is the predominant post-translational modification of mammalian αS. Using circular dichroism, isothermal titration calorimetry, and fluorescence spectroscopy, we have analyzed the effects of N-terminal acetylation on the propensity of recombinant human αS to form the two conformational states in interaction with lipid membranes. Small unilamellar vesicles of negatively charged lipids served as model membranes. Consistent with previous NMR studies using phosphatidylserine, we found that membrane-induced α-helical folding was enhanced by N-terminal acetylation and that greater exothermic heat could be measured upon vesicle binding of the modified protein. Interestingly, the folding and lipid binding enhancements with phosphatidylserine *in vitro* were weak when compared to that of αS with GM1, a lipid enriched in presynaptic membranes. The resultant increase in helical folding propensity of N-acetylated αS enhanced its resistance to aggregation. Our findings demonstrate the significance of the extreme N-terminus for folding nucleation, for relative GM1 specificity of αS-membrane interaction, and for a protective function of N-terminal-acetylation against αS aggregation mediated by GM1.

## Introduction

The conformational plasticity of α-synuclein (αS), a cytosolic protein abundant in neurons, erythrocytes and endothelial cells [1], has been associated with differential physiological and pathophysiological properties. Intraneuronal aggregates consisting of αS in a β-sheet-rich conformation, called Lewy bodies and neurites, are a pathological hallmark of Parkinson’s disease (PD) and several related human brain disorders.

We and others recently reported that αS unbound to lipids can exist in the cytosol in an α-helical, oligomeric state that is much more resistant to aggregation than the unfolded monomer [2]–[4]. The exact folding and oligomerization pathway that leads to these helical oligomers *in vivo* is not known. The induction of α-helix formation of αS in its unfolded monomeric state *in vitro* has been shown to be mediated *inter alia* by its binding to small unilamellar vesicles (SUV) composed of negatively charged lipids [5]; lipid binding and folding leads to protection of αS from pathology-associated misfolding [6], which suggests the existence of a beneficial folding pathway of the physiological form of the protein. Lipid interaction has been shown to be particularly strong for the neuronal monosialoganglioside, GM1 [7]. It also has been reported that the extreme N-terminus of αS is important for lipid interaction and membrane-induced helix formation [8]–[10].

Recent reports indicate that all detectable αS *in vivo* is post-translationally modified by an acetyl group attached to the α-amino group of the first N-terminal amino acid [2], [11], [12]. Despite this knowledge, most *in vitro* studies of αS have been performed on recombinantly expressed protein lacking this physiologically meaningful modification. N-alpha-acetylation (NAA) has been implicated in stabilizing helical conformations among the ensemble of unfolded αS conformations *in vitro* [13] as well as leading to the formation of folded, oligomeric αS [14], which resembles the recently discovered aggregation-resistant forms of native αS isolated from cells [2], [15]. Given the apparent importance of NAA for the conformational landscape of αS and its interaction with lipids, we performed systematic biophysical analyses of the impact of the acetyl group on the thermodynamics of αS folding and lipid binding, and we explored how the pathology-associated properties of αS may be affected. We employed isothermal titration calorimetry in tandem with CD spectroscopy to dissect the thermodynamic parameters associated with membrane binding and the concomitant coil-helix transition of αS with versus without N-terminal acetylation. We found that the N-terminal acetyl group enhanced general membrane binding and mediated a relative specificity of αS for the neuronal ganglioside GM1. Interestingly this enhanced interaction led to a complete resistance to fibrillar aggregation of acetylated αS but not of the non-acetylated form, as shown by fluorescence spectroscopy.

## Materials and Methods

### Materials

1-palmitoyl-2-oleoyl-sn-glycero-3-phospho-L-serine (POPS), 1-palmitoyl-2-oleoyl-sn-glycero-3-phosphocholine (POPC), monosialoganglioside GM1 and GM3 (both bovine) were purchased from Matreya LLC (Pleasant Gap, PA) as dry powder. All other chemicals were obtained from Sigma-Aldrich unless otherwise noted.

### Protein Preparation

A construct based on the sequence of human αS in pET5a was used to transform Escherichia coli BL21(DE3). For N-acetylated αS, the bacteria were co-transformed with a pACYCduet plasmid containing cDNA encoding for both catalytic (Naa20) and regulatory (Naa25) subunits of the fission yeast NatB complex, a generous gift by Daniel Mulvihil (University of Kent, UK). Bacterial cultures were induced with isopropyl-β-D-thiogalactopyranoside for 4 h, and lysed by sonication in 20 mM Tris buffer, pH 8.0, 25 mM NaCl. The lysate was quickly microwaved to boiling in order to denature common proteases, and then kept at 65°C for 30 min to precipitate heat unstable proteins quantitatively. The supernatant of a 20 min 20,000×g spin was further processed. For non-denaturing purification, the boiling step was replaced by a 50% (NH4)2SO4 precipitation step, and the pellet was taken up in 20 mM tris buffer, pH 8.0, 25 mM NaCl. The sample was injected onto a 5-ml HiTrap Q HP anion exchange column (GE Healthcare, Pittsburgh, PA), equilibrated with 20 mM Tris buffer, pH 8.0, 25 mM NaCl. αS was eluted from the column with a 25–1000 mM NaCl gradient in 20 mM Tris buffer, pH 8.0. Peak fractions were pooled and further purified via gel filtration on a Superdex 200 column (GE Healthcare, Pittsburgh PA) with 50 mM ammonium acetate pH 7.4 as running buffer. The peak fractions were lyophilized and N-terminal acetylation was validated by mass spectrometry.

### Isolation of αS from human erythrocytes

The purification was conducted as described previously [2]. For a detailed description please see http://www.nature.com/protocolexchange/protocols/2136.

### CD spectroscopy

Spectra were recorded with a Jasco 815 spectropolarimeter (Jasco Inc., MD). A cuvette with 1 mm path length was filled with 200 µL of the protein solution. The proteins were titrated with phospholipid vesicles using a micro pipet with gel loading tip for lipid addition and mixing of the protein and vesicle solutions. Control spectra obtained with titrations of vesicle suspensions in buffer alone were subtracted from the experimental spectra. Temperature control with an accuracy of +/−0.5°C in the cuvette was achieved with a heating/cooling accessory using a Peltier element. The helicity of the protein (i.e. the percentage of the entire protein sequence in an α-helical state) was obtained from the mean residue ellipticities, Θ_222_, according to f_helix_ = (Θ_222_– Θ_coil_ )/( Θ_coil_ – Θ_helix_) where % helicity = 100 f_helix_. Here the mean residues ellipticities at 222 nm for the completely unfolded and completely folded peptides were obtained from Θ_coil_ = 640–45T/°C and Θ_helix_ = –40000(1–2.5/n) + 100T/°C, where n is the number of amino acids in the polypeptide [16].

### Isothermal Titration Calorimetry

Isothermal titration calorimetry measurements were performed with an iTC200 instrument (MicroCal, Amherst, MA). Typically, 18 lipid suspension injections of 2 µL each (+1 pre-injection with 0.4 µl) were titrated into the calorimeter chamber containing 200 µL of the protein solution. Control injections of vesicles into buffer alone were performed and used for baseline subtraction. The data were processed using the MicroCal ORIGIN 5.0 software, and thermodynamic parameters were determined from the sigmoidal titration curves with the assumption that there are independent saturable binding sites in the vesicle interface. The ligand concentration was equated with the total lipid concentration in the syringe; the aggregation state of the lipids and the accessibility of the inner lipid bilayer of the unilamellar vesicles were neglected. *N* independent lipid binding sites on the protein were assumed. Incremental heat values Δ*Q* = *Q*_i-1_− Q_i_ were calculated and the experimental data was fitted by variation of *N* and the microscopic binding constant *K*_b’_, as well as the microscopic enthalpy change Δ*H*’ according to the standard Marquard-Levenberg algorithm [17]. This yields the enthalpy per mole of protein, and a single macroscopic binding constant *K*_b_ through Δ*H* ^0^ = *N*Δ*H’* and *K*_b_ = *NK*_b_’, as well as the free energy (Δ*G* ^0^ = −*RT* ln *K*_b_) and entropy (Δ*S* ^0^ = (Δ*H* ^0^ − Δ*G* ^0^)/*T*) of the binding reaction with respect to the protein.

### Vesicle Preparation

Mixtures of phospholipids were first taken up and mixed in tert-butanol. After overnight lyophilization, the lipid mixtures were hydrated for 30 min at 25°C in 1 mL of buffer containing 10 mM ammonium acetate, pH 7.4. Small unilamellar vesicles were prepared by pulse-sonicating the phospholipid suspensions for 20 min at 4°C with a microtip sonicator (Fischer Sonic Dismembrator Model 300, Thermo Scientific, MA, USA). The translucent vesicle solutions were spun down at 15,000×g for 10 min to pellet any remnants of solid metal particles. Quality control of the vesicles was performed using a Malvern Dyna Pro dynamic light scattering instrument (Westborough, MA), which showed uniform vesicles with a mean diameter of about 30 nm.

### ThT fluorescence assay

α-Synuclein fibrils were formed by incubating 0.6 mg/ml protein in the presence of 50 mM ammonium acetate at pH 7.4. Aliquots of 2.5 µL of the solution were added to 197.5 µL of 10 µM thioflavin T in 10 mM glycine at pH 9.0 [18], and the fluorescence intensity was recorded at 485 nm with excitation at 447 nm in a Synergy H1 plate reader (Biotek, Winooski, VT).

## Results

### Variable changes in αS secondary structure upon N-acetylation

**Figure 1:**
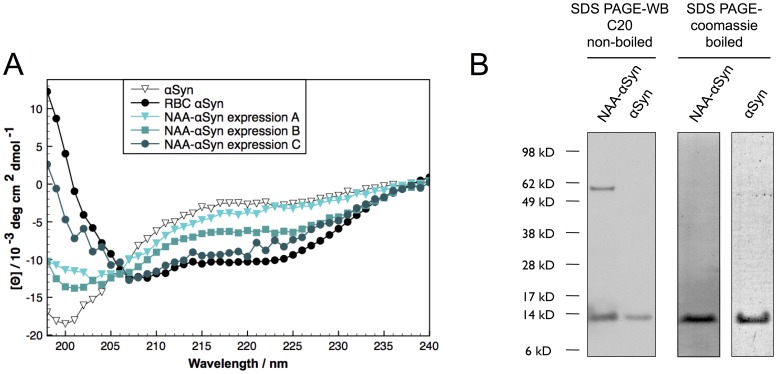
Variability in the folding of purified recombinant NAA-αS under non-denaturing conditions. **A:** CD spectroscopy of bacterially expressed and purified NAA-αS and αS under non-denaturing conditions. No variability in the secondary structure was seen with unmodified αS. In contrast, NAA-αS showed batch-to-batch variability in folding of the obtained preps. **B:** SDS-PAGE Western blot and Coomassie stain of αS and NAA-αS (“expression C”). The samples were not boiled in SDS-sample buffer before gel loading for Western blotting. The NAA-αS banding pattern indicates some SDS-stable tetrameric protein present in the sample, while no higher bands could be detected in non-acetylated αS. For coomassie staining, the samples were boiled in sample buffer, leading to a single stainable band at 14 kDa, indicative of the high purity of the sample.

We recently discovered the existence of a helically folded oligomeric form of endogenous αS in human cells, an observation that has led to an ongoing controversy in the field. The main origin of this controversy is that the protein had previously been characterized as “natively unfolded”, based principally on studies of recombinantly expressed αS purified from bacterial lysates. This seemed to disagree directly with our characterization of the protein isolated under non-denaturing conditions from human cells. A major difference between αS obtained from these two sources is the presence of N-terminal alpha acetylation (NAA) in the human protein, a post-translational modification found commonly in mammalian proteins. We therefore first examined the effect of NAA on the overall folding of bacterially expressed αS in the absence of structure-inducing detergents or lipids, in comparison with αS isolated from fresh human red blood cells (RBC-αS). To this end, we co-expressed αS in bacteria with the fission yeast complex N-acetyltransferase B (NatB), which is reported to cause targeted NAA of other recombinantly expressed proteins that share with αS the N-terminal amino acid motif Met-Asp (MD) [19]. In order to preserve any structure induced by the NAA, we used a non-denaturing protocol to purify the bacterially expressed recombinant protein. This method generated preparations that were 100% N-α-acetylated αS (NAA-αS), as confirmed by mass spectrometry (data not shown). As shown by circular dichroism (CD) spectra, we obtained some batches of α-helically folded NAA-αS in the absence of detergent (e.g. the BOG used by Trexler et al. [14]) or lipids (Fig. 1A), and such preparations contained minor amounts of SDS-stable oligomeric NAA-αS (Fig. 1B), in agreement with a recent report by Trexler et al. [14] and closely resembling endogenous RBC-αS. However, in other batches ostensibly prepared identically under non-denaturing conditions, we observed mainly unfolded NAA-αS, similar to the NAA-αS preparations reported by Fauvet et al. [20], and yet other preparations were folded in an intermediate state (Fig. 1A). Even the maximally unfolded preparations had a slightly higher α-helical content than comparable preparations of αS obtained without co-expression of NatB (approximately 9% vs 5% helicity measured by CD spectroscopy). This increased helicity could not be prevented by application of heat denaturation (data not shown) and thus appeared to be an intrinsic property of NAA-αS independent of the purification method. It is important to note that no such variability in the amount of helical content was observed for bacterial preparations of αS without N-acetylation under non-denaturing conditions; it was all unfolded with only minor helical content (5%). The cause(s) of the conformational variability in the recombinant batches of NAA-αS prepared under seemingly identical conditions are under investigation but currently remain elusive. None of the variations in the temperature during IPTG induction (25 or 37°C), cell density at harvest (OD_600_: 0.8–1.5) or the IPTG concentration used for induction (0.1–1 mM) had a clear correlation with helicity. It has to be noted though that preparations with higher helical content typically had lower overall protein yield and that N-terminally acetylated αS seemed to be more resistant to N-terminal degradation during the purification procedure in general. Given the lack of control over batch variability under non-denaturing conditions, we decided to analyze separately the properties of the invariably mostly unfolded NAA-αS obtained under denaturing conditions in regards to its interaction with lipids in order to gain more insight into possible folding mechanisms of this post-translationally modified form of αS. We therefore used a heat denaturation step in our purification protocol for both NAA-αS and non-acetylated αS in all experiments described below to obtain maximally unfolded protein preparations. This denaturation step allowed us to control variability in helical content and assess the impact of N-terminal acetylation on initiation of in vitro folding upon in interaction with lipids.

### Folding Initiation of NAA-αS by Negatively Charged Lipid Vesicles

**Figure 2:**
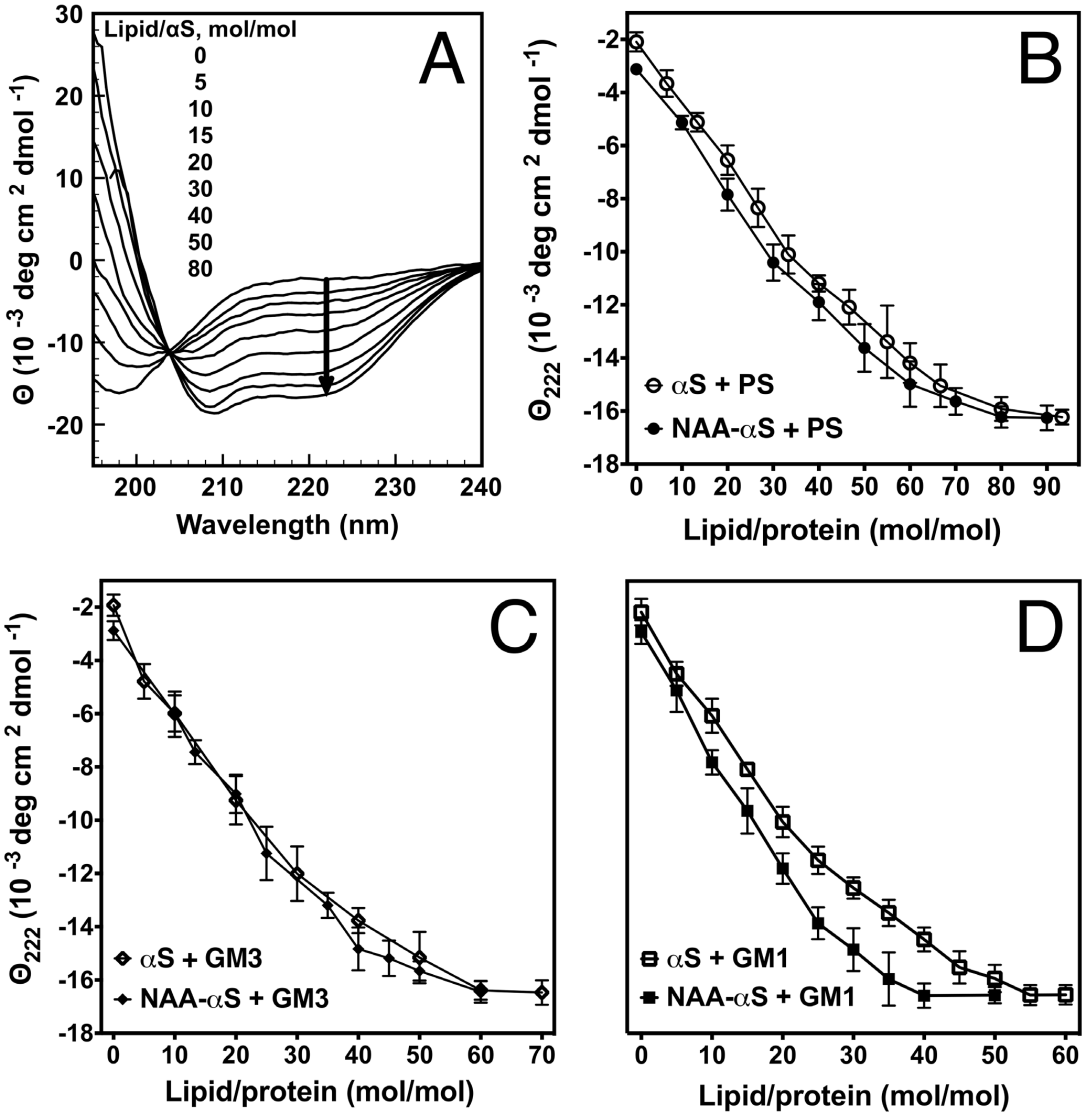
Structural change induced in αS vs. NAA-αS by negatively charged vesicles. **A:** Example of CD spectroscopy of PS vesicles titrated into NAA-αS solutions at 25°C. Spectra are shown at ascending lipid/protein ratios. Note the isobestic point (at 204 nm) indicative of a pure two state coil-helix transition. The arrow indicates the decrease in ellipticity at 222 nm as a consequence of helical folding. B–D: CD spectroscopy measurements of αS (5 µM) titration with PS (POPC/POPS 4∶1, 40 mM) (**B**), GM3 (POPC/GM3 4:1, 40 mM) (**C**) and GM1 (POPC/GM1 4:1, 40 mM) (**D**). Decrease in MRE at 222 nm upon titration of αS with PS, GM3 and GM1 containing vesicles correlates with increased α-helical structure in either NAA-αS or non-acetylated αS.

Lipid vesicles bearing a net negative surface charge have been reported to bind monomeric αS associated with a concomitant conformational transition from random coil to α-helix [5], [7]. Such vesicles were therefore chosen to study the impact of the NAA modification on helix formation. Three model lipids were chosen: phosphatidylserine (PS), for its abundance in neuronal membranes [21] and to compare the resultant data to previous studies [13]; GM1 ganglioside, for its reported specific interaction with αS [7]; and GM3 as a control lipid to test the specificity of N-acetylation for the headgroup of GM1. We prepared mixtures of each of these lipids (20%) with phosphatidylcholine (PC, 80%) due to the lack of interaction of αS with PC [22] and the inability of PS or GM1 to form stable small unilamellar vesicles (SUV) on their own. We tested for structure induction by titrating increasing amounts of the lipids into the αS protein solutions (typically 5 µM) and assessing conformational changes by far-UV circular dichroism (CD) spectroscopy. In tandem, we detected the thermodynamic changes by isothermal titration calorimetry (ITC) with identical protein concentrations and lipid mixtures. Figures 2A and B show titrations of PS-containing POPC vesicles (POPC/POPS 4∶1) into protein solutions of NAA-αS measured by CD. The coil-helix transition can be conveniently followed by spectroscopic measurement of CD at a wavelength of 222 nm. As seen in Fig. 2A, a decrease of the ellipticity (arrow) is indicative of an increasing conformational change towards α-helical secondary structure; this characteristic allows us to readily compare the induction of secondary structure for αS by plotting the decrease of ellipticity at 222 nm for a given αS/lipid ratio. To account for slight differences in αS concentration between measurements, we show here the measured CD signal as the mean residue ellipticity (MRE, Θ).

In Figs. 2B, C and D, the CD titration curves for αS and NAA-αS during interaction with PS, GM3 or GM1 are shown. The initial slopes of the MRE versus the lipid/protein molar ratio plots for PS titration into solutions of αS vs. NAA-αS are nearly identical, suggesting that their interaction with the negatively charged headgroup of PS is very similar (Fig. 2B). While a small difference in average MRE between αS and NAA-αS can be detected for a given PS concentration, this is also seen in the absence of lipids; this result indicates that there is a higher general baseline of α-helical content for NAA-αS. The titration endpoints where maximum ellipticity is achieved converge, implicating a very similar amount of maximal secondary structure (approx. 44% α-helix; see Methods) at a PS-to-synuclein ratio of 80∶1 for both NAA-αS and αS. This value is almost identical to the approx. 46% helical content that can be found in natively purified human RBC-αS tetramers [2]. This result in agreement with previous studies by Maltsev et al. [13] and Dikiy et al. [23], but the differences seem small when compared to experimental variability seen in between different measurements. Interestingly, titrations with vesicles containing the gangliosides GM3 and GM1 showed a decreased barrier to folding initiation for both forms of αS when compared to PS (Figs. 2C and D) and thus a much stronger effect of N-acetylation was observed than before for PS. Since all three lipid types have the same net charge, the results imply that the folding nucleation by the interaction of the αS N-terminus with the gangliosides is specifically strengthened, possibly through hydrogen bonds between the acetylated protein and the sugars in the headgroups. Both αS and NAA-αS achieved maximum folding at lipid-to-protein ratios of about 60∶1 when titrated with GM3 vesicles. However, titrations with the negatively-charged ganglioside GM1 gave a significantly steeper slope for NAA-αS than αS, suggesting an increased conformational sensitivity to lipid contact by NAA-αS. In the presence of GM1 containing SUVs, the ellipticity of NAA-αS reaches a constant value at lipid/protein ratios of 40∶1, while a higher ratio (55∶1) is needed for maximum structure induction in non-acetylated αS (Fig. 2 D). As the only structural difference between GM1 and GM3 can be found in the amount of sugar residues attached to the negatively charged sialic acid, this directly points at an interaction of the N-acetyl group of the protein with the oligosaccharide chain of the lipid, which leads to a decreased activation barrier for helical folding.

### Impact of N-Acetylation on the Thermodynamics of Lipid Interaction

To assess quantitatively the thermodynamics of the lipid-αS interaction, we employed ITC. ITC offers many advantages over other techniques because the heat signal measured is a nearly universal property of chemical binding reactions as well as of the physical adsorption processes. ITC provides the opportunity to not only measure accurately the binding constant of the protein-lipid association, but also dissect the contributions of thermodynamic parameters such as enthalpy or entropy to the process of adsorption and folding.

**Figure 3:**
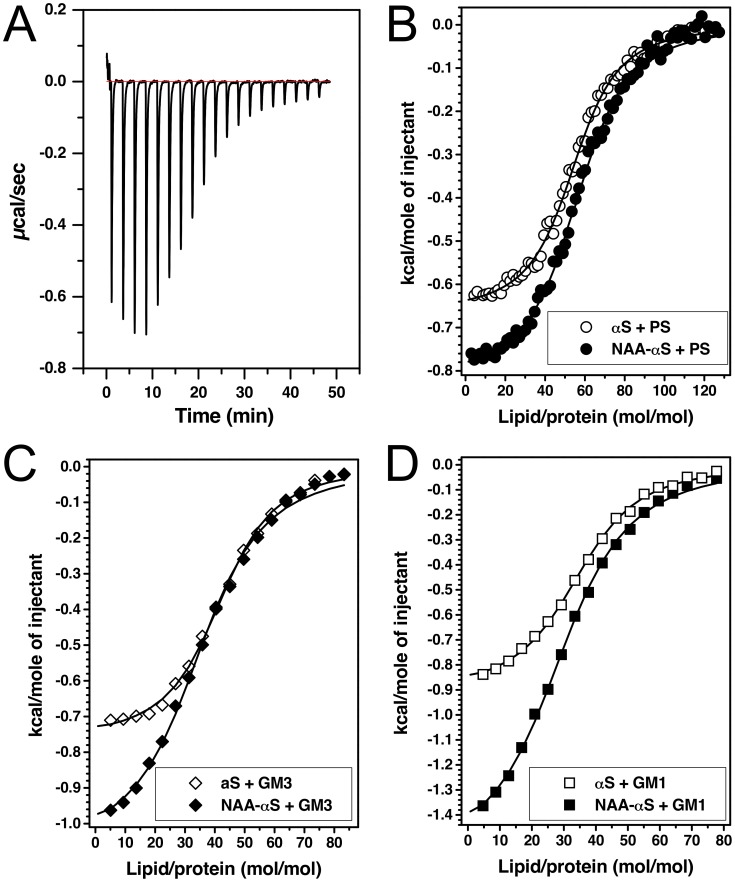
ITC-measured heat release curves of NAA-αS and αS upon titration with negatively charged lipid vesicles. **A:** Example of primary heat signal obtained from titration calorimetry of PS vesicles titrated into solutions of NAA-αS at 25°C measured in tandem with the CD spectra in Fig. 2. **B:** Integrated heat signals of titrations of PS vesicles (POPC/POPS 4∶1, 40 mM) into protein solutions of NAA-αS and αS (5 µM). The sigmoidal titration curve suggests simple protein-lipid binding with saturable binding sites. **C:** Titrations of GM3 vesicles (POPC/GM3 4:1, 40 mM) into solutions of NAA-αS and αS (5 µM) at 25°C. **D:** Titration of GM1 vesicles (POPC/GM1 4:1, 40 mM) vesicles into the two protein solutions (5 µM). Note the doubled heat release for NAA-αS compared to non-acetylated αS. All measurements were conducted at 25°C.

Fig. 3A shows aliquots of PS SUVs titrated into a calorimeter cell containing solutions of αS. The negative heat-exchange upon vesicle injection shows that the lipid-protein interaction is exothermic. After integration of the obtained heat signals, a binding or partition curve can be derived. As seen in Figs. 3B–D, the titration curves show a classic sigmoidal shape reminiscent of what would be observed in a specific ligand-protein interaction, not a partition equilibrium that one would expect in simple membrane insertion of a polypeptide. Thus, we assumed the presence of uniform saturable lipid binding sites on both αS and NAA-αS and evaluated the data accordingly (see Methods). We summarize all of the biophysical parameters obtained by ITC and CD in Table 1.

**Table 1:** Thermodynamic and structural parameters for NAA-αS and αS upon SUV titration.

	ΔH_0_ kcal/mol	K_B_ ×10^6^ M^-1^	ΔS_0_T kcal/mol	ΔG_0_ kcal/mol	Helicity %	Bound lipids/αS
**αS**	n/a	n/a	n/a	n/a	5 (10)	n/a
**+ PS**	−37.2+/−1.3	4.6+/−1.2	−28.1+/−1.9	−9.1+/−0.5	44 (95)	56+/−5
**+ GM3**	−31.3+/−2.5	5.8+/−1.3	−22.0+/−3.6	−9.2+/−0.7	44 (95)	41+/−6
**+ GM1**	−28.2+/−2.7	0.8+/−1.9	−20.2+/−5.6	−8.8+/−0.5	44 (95)	31+/−5
**NAA-αS**	n/a	n/a	n/a	n/a	9 (19)	n/a
**+ PS**	−48.4+/−3.4	4.0+/−2.3	−39.4+/−3.1	−9.0+/−0.5	43 (95)	59+/−4
**+ GM3**	−38.6+/−5.1	2.8+/−2.4	−29.8+/−5.9	−8.8+/−0.4	44 (95)	36+/−8
**+ GM1**	−55.2+/−6.5	1.23+/−2.1	−46.9+/−6.4	−8.3+/−0.2	45 (96)	35+/−8

Experiments were performed at 25°C. Depicted values are means values from 3 independent measurements with standard deviations shown for the ITC measurements. Helicity values are means from 3 independent measurements.

The fitting results for PS vesicles indicate a comparable binding constant (K_B_) and hence comparable free energy (**Δ**G_0_) for αS and NAA-αS, demonstrating a very small effect size of N-terminal acetylation, in agreement with recent literature [13], [23]. However this comes as a surprise given that slightly lower binding for NAA-αS was expected due to the elimination of the positive charge at the alpha-amino group by acetylation and the resultant slight decrease in the electrostatic interaction between the positively charged N-terminus of the unmodified protein and the negatively charged headgroup of the lipid. The calculated total enthalpy of the lipid-protein interaction for both forms of synuclein is approximately 0.4–0.5 kcal/mol per amino acid residue of the lipid binding N-terminal region aa1–94 [24], which is in the expected range for membrane insertion and secondary structure formation [25]. The contributions of enthalpy (**Δ**H_0_) and entropy (ΔS_0_) are similar for αS and NAA-αS, with the lipid interaction of NAA-αS being more exothermic (Table 1). Both interactions are enthalpy driven and are counterbalanced by a loss in entropy, probably due to the formation of secondary structure and restricted diffusion of lipids around the protein binding sites. This effect is larger for NAA-αS than for αS, indicating a slightly larger amount of helical conformation in NAA-αS, as seen in the respective CD spectra in Figures 2B and C. The inflection point of the titration curve at a lipid/protein ratio of 70∶1 is in agreement with the CD data, showing a beginning saturation of protein binding accompanying helix formation.

These differences are again exaggerated in the interaction with GM3. As can be seen from Figs. 3C and D, the interaction of αS with ganglioside in general leads to greater heat release compared to the titration with PS. The more exothermic reaction of NAA-αS points to an increased interaction of the acetyl group with GM3 and PS. The gain of heat release in the presence of NAA-αS is comparable between GM3 and PS, however indicating a lack of specificity of the N-acetylation for either of these lipids. As the position and slope of the inflection point of the binding curves is seemingly identical between NAA-αS and αS, the increased heat release is not attributable to a larger amount of bound lipid or tighter binding in general. It could be assumed that the additional heat release is due to increased structure formation in either the protein or the lipid membrane.

As shown in Fig. 3D, the titration curves for GM1-containing vesicles show a markedly different thermodynamic profile between αS and NAA-αS. While the calculated free energy (ΔG_0_) and binding constants (K_B_) are closely similar within the limits of experimental variation (Table 1), the total enthalpy (ΔH_0_) measured upon NAA-αS-GM1 interaction is approx. −55 kcal/mol, about twofold of the 28 kcal/mol detected for non-modified αS. This strong exothermic process is compensated by a loss in entropy (ΔS_0_T), which is again twofold larger for NAA-αS than for αS. Analysis of the inflection points of both titration curves indicates that a similar number of GM1 molecules are bound to NAA-αS and αS, although the number is slightly higher for the acetylated form (35 for NAA-αS vs. 31 for αS). Given the small contribution of the electrostatic interaction to the total enthalpy [26] this slight increase in the average in bound lipid should not account for the entire twofold change in enthalpy and entropy.

**Figure 4:**
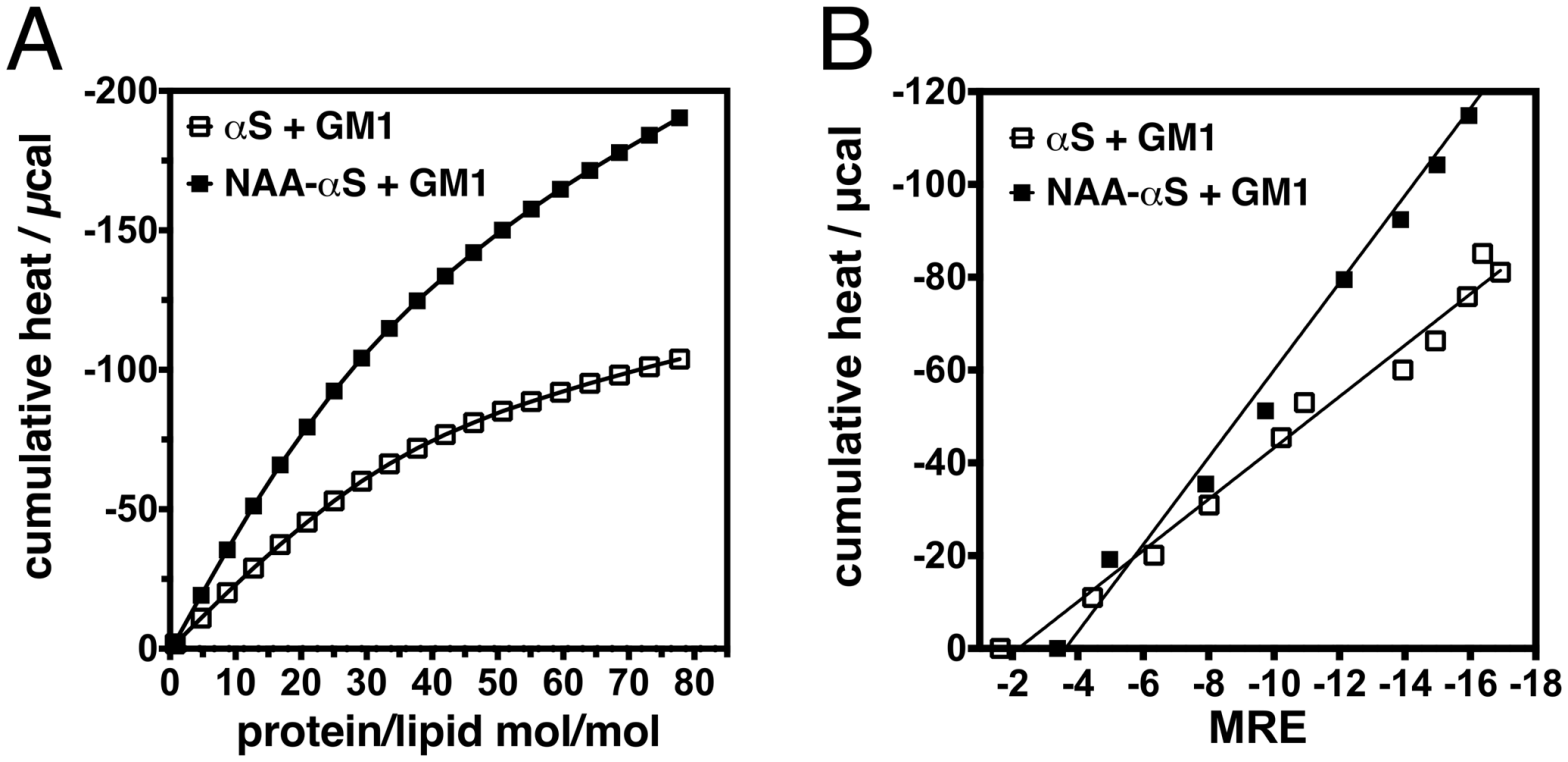
Characterization of synuclein-GM1 interaction. **A.** Cumulative reaction enthalpy of αS vs. NAA-αS (each at 5 µM) upon vesicle titration with GM1 vesicles is shown as a function of lipid/protein ratio. Experiments were conducted at 25°C. Note the strongly increased heat release for NAA-αS upon titration with GM1. **B.** ITC-CD correlation plots showing the different interactions of NAA-αS and αS with GM1 vesicles. The interaction of NAA-αS and αS with GM1 vesicles is shown as correlation of MRE at 222 nm (abscissa) with corresponding cumulative heat release (ordinate). The greater slope obtained for NAA-αS indicates an increased heat release accompanied by similar secondary structure induction.

The increased heat release upon lipid binding can be easily visualized when the cumulative obtained heat is plotted against the amount of titrated lipid. As seen in Fig. 4A, each single lipid titration leads to an increased exothermic reaction when the N-terminal acetyl group is present. This is surprising, as both forms of the protein have similar lipid binding constants. However, the heat measured by ITC must be treated as a composite quantity that summates interfacial binding and structure formation in the protein. A linear relationship was found between the cumulative heat released by vesicle binding and the MRE at 222 nm, which indicates that the energy transfer is associated with an equivalent increment in secondary structure formation (MRE) (Fig. 4B).

While the correlations of titrations with PS and GM3 vesicles were almost identical for both αS variants (data not shown), in the case of GM1, the slopes of the linear regression lines are almost twice as great for NAA-αS than for αS. The MRE reaches similar values for all 3 lipids and thereby demonstrates a very similar helix formation. This phenomenon suggests that the large exothermic heat observed upon NAA-αS-GM1 interaction cannot be solely explained by the electrostatic binding of αS to the membrane vesicle surface and the accompanying coil-helix transition. The larger release of exothermic heat with a decrease in entropy must rather be accounted for by structure induction other than α-helix formation in the protein. This could be through increased clustering of GM1 lipids around the bound protein or additional tertiary or quaternary structure formation of NAA-αS at the membrane interface. The additional formation of structure would also explain the larger comparative loss of entropy that accompanies lipid interaction (−47 and −20 kcal/mol for NAA-αS and αS, respectively).

### Inhibition of αS Aggregation by both N-Acetylation and GM1 Interaction

**Figure 5:**
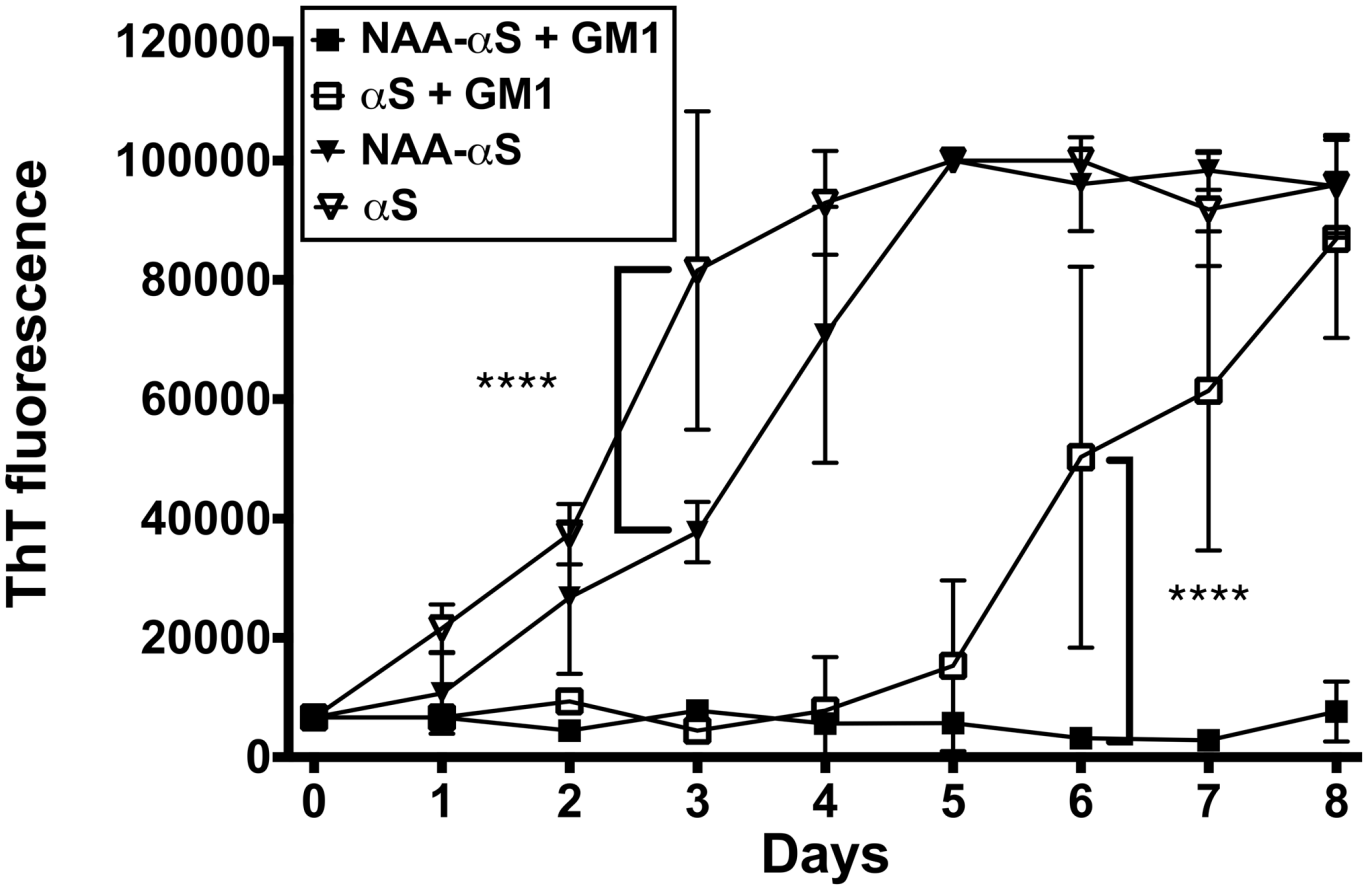
N-alpha acetylation has a functional impact on αS aggregation into amyloid fibrils. Aggregation of NAA-αS and αS (0.6 mg/ml) in the absence or presence of GM1 containing vesicles (GM1/protein 10∶1 mol/mol) measured by an increase in thioflavin T fluorescence. Standard deviations are calculated from N = 4 experiments. The presence of N-acetylation or GM1 vesicles binding each led to increased resistance to aggregation, with the effects being cumulative. A 2-way ANOVA test was used to test for significance at selected time points. **** = p<0.0001.

Inhibition of αS β-sheet-fibrillation by lipid vesicles [6] and the presence of helical secondary structure [2], [3] have been reported to be potential modulators of pathogenic αS aggregation; this suggests that the slightly higher helicity of NAA-αS can mediate resistance to aggregation. We agitated NAA-αS or αS at a concentration of 0.6 mg/ml at 37°C, conditions that had been optimized to detect small changes in aggregation propensity while maintaining manageable time frames. In order to asses the amount of helix induction and amount of protein bound to lipid under these exact conditions, we repeated CD and ITC measurements with both NAA-αS and αS for GM1 at 37°C, obtaining K_B_ and MRE values identical to experiments conducted at 25°C (data not shown). Using a thioflavin T aggregation assay, we monitored the aggregation for 8 days. The fibrillation of NAA-αS and αS were measured as an increase in fluorescence intensity of the thioflavin T dye upon binding to β-sheet-rich aggregates that had formed in the samples over time. As seen in Fig. 5, pure protein solutions of NAA-αS displayed slower aggregation kinetics compared to those of pure αS as reported in the literature [27] and expected from higher helical content of the sample. To test the effect of lipid association in combination with acetylation, we conducted experiments in parallel in the presence of POPC/GM1 SUV at a protein/lipid ratio of 1∶10, a molar ratio that only induces a minor increase in helical structure of both NAA-αS and αS (Fig. 2). The incubation with GM1 vesicles led to a delay from 4 to 8 days in the time necessary for maximal fibril formation of non-acetylated αS in accordance with previous studies [7]. This effect was more pronounced with NAA-αS in that the addition of GM1 SUV seemingly led to a complete prevention of thioflavin-positive aggregation, at least for the 8 days of the experiment (Fig. 5). A possible explanation for this cumulative effect of GM1 interaction and acetylation could be the small but significant increase in helicity measured under these conditions for NAA-αS when compared to non-acetylated αS (Fig. 2). This would indicate that even a small population of GM1-bound αS can contribute to stabilizing helical conformations that raise the energetic barrier for refolding into beta-sheet and thus protects the whole ensemble of different conformations that must occur in solution due to rapid exchange. Alternatively, a small difference in binding affinity detected for NAA-αS and GM1 (Table 1) could lead to a slightly smaller concentration of non-lipid bound NAA-αS which could slow self-association in the free form of the protein.

## Discussion

We and others recently reported the existence of an abundant tetrameric form of αS in human cells [2], [4] and in an N-terminally extended recombinant construct [3] that is helically folded and relatively resistant to amyloid aggregation. This finding was in stark contrast to numerous reports characterizing recombinantly expressed αS as a “natively unfolded” monomer (e.g. [28]). The finding has led to studies either disputing it [29] or independently reproducing our findings in human erythrocytes [15] or in some part in murine brain tissue [30]. The presence of N-alpha-acetylation in endogenous human αS then became a focus of study, since it seemed to be the principal post-translational modification (PMT) distinguishing recombinantly expressed and endogenous cellular αS. One study showed that this PMT alone can be responsible for the formation of helically-folded oligomers under certain conditions [14]. While we were able to find some evidence for the latter finding in that we isolated some helically folded and apparently tetrameric αS from bacteria expressing αS plus N-acetyl-transferase complex B (Fig. 1), our findings imply that N-acetylation alone is necessary but insufficient for the formation of helical tetramer. The variability in this result led us to investigate systematically the effect of N-acetylation on αS folding initiation. By studying the lipid assisted folding of αS, we may thereby gain more insight into the potential mechanism of helical tetramer formation in vivo. Although recent studies have been investigating the impact of N-acetylation on helical folding of αS in the presence of lipid, observing slightly increased interaction with phosphatidylserine [13] in agreement with our data, the strong and apparently specific effect of the interaction of the extreme N-terminus with GM1 ganglioside has not been reported heretofore.

Prior work demonstrated the ability of αS to bind small unilamellar lipid vesicles, with a concomitant random coil-to-helix transition when in contact with negatively charged lipids [5], [31], [32]. This lipid interaction has been associated with stabilization of vesicular plasticity and the control of vesicle fusion [33]–[36] as well as the inhibition of fibril formation [7]. Such results suggest the importance of mutual structure stabilization of membrane and protein in normal cellular function. Our new results here support the increased propensity of αS toward lipid binding and helical folding upon N-acetylation, a post-translational modification that seems to be present in the large majority of endogenously expressed αS molecules in human tissue [2], [11], [12]. Starting with phosphatidylserine, the model lipid classically used to induce helical folding in αS [5] and used to test the effect of N-acetylation [13], we probed for differences in lipid binding and secondary structure formation.

Phosphatidylserine showed little preference for interacting with NAA-αS over the non-acetylated protein. Whereas titrations of PS vesicle solutions showed minimal but measurable effects of N-terminal acetylation on alpha-helix induction, an analysis of the thermodynamic parameters by ITC revealed a slightly elevated heat release for NAA-αS. This is in agreement with previous studies that also recorded a small but detectable effect of N-terminal acetylation upon interaction with PS. These results are surprising, given that the removal of a positive charge from the extreme N-terminus seems not to lessen but to strengthen the interaction with the negatively charged membrane surface. The same was generally true for the ganglioside GM3, even though the interaction with the sugar headgroup led to a much greater exothermic heat release and increased folding initiation for both αS and NAA-αS when compared to PS. This could indicate a better hydrophobic match between the now less charged N-terminus and the acyl chains of the lipid, possibly by a deeper intrusion of the amphipathic helix into the hydrophobic core of the bilayer [37].

Interestingly, we show here that the N-acetylation seems to specifically strengthen the interaction between NAA-αS and GM1. Not only does the N-terminal acetylation apparently promote secondary structure formation upon GM1 binding, but the thermodynamics of the binding event are also altered by the presence of the acetyl group, leading to an almost twofold increase in heat release of NAA-αS over unmodified αS.

Aggregation of αS into amyloid fibrils has been centrally implicated in the pathogenesis of Parkinson’s disease. In this respect, we find that the enhanced helical-folding propensity of NAA-αS leads to enhanced aggregation resistance, with fibril formation being undetectable by our Thioflavin T fluorescence assay in the presence of GM1. αS can be found in lipid raft and raft-like regions of the synapse, where it co-localizes with GM1 [38] and from which it can be released during synaptic activity [39]. We speculate that binding to GM1-rich membranes could therefore protect monomeric αS from pathogenic aggregation despite its leading to a higher local concentration of αS at the membrane interface. It is interesting to note that while the familial PD-associated αS mutations A53T and A30P have been reported to accelerate aggregation [40], [41], the mutation A30P has also been reported to disrupt membrane interaction and helix folding [42]. Considering membrane binding and helix formation as a possible protective mechanism, we suggest that N-terminal acetylation and the A30P mutations may work in opposite directions; while A30P accelerates disease relevant mechanisms, NAA decelerates it, especially in the presence of GM1 ganglioside.

An extensive body of literature can be found on the potential involvement of GM1 gangliosides in the pathogenesis of PD [43], through either accelerated αS aggregation in mice having decreased GM1 levels [44], [45] or a decrease in PD-type symptoms upon administration of GM1 [46], [47]. There is also evidence for a connection between Parkinson’s disease and disorders with defective glucocerebroside metabolism like Gaucher’s disease that indirectly affect GM1 levels [48]. Our findings here could provide an explanation for an apparent correlation between the presence of excess GM1 and the moderation of synuclein-dependent neuronal dysfunction.

In summary, our data suggests that the overall effect of the N-terminal acetylation should not be neglected in studying the function or biochemical/biophysical properties of αS. Given the apparent absence of non-acetylated αS in mammalian tissues [2], [11], [12], we believe it is imperative to use NAA-αS in future *in vitro* studies, in order to reduce artifacts arising from recombinant expression and not miss any meaningful changes in the highly complex folding landscape of this disease-relevant protein.
